# Supplementary material for: Causal Relationship between Aging and Anorexia Nervosa: A White-Matter-Microstructure-Mediated Mendelian Randomization Analysis
Source: Biomedicines. 2024 Aug 16;12(8):1874. doi: 10.3390/biomedicines12081874 (PMC11351342; doi:10.3390/biomedicines12081874)
Supplement: Supplementary file 1 [file biomedicines-12-01874-s001.zip › Supplementary Figure/Figure S8/Leaveoneout plot of MR analysis for TL to ubm-b-2089.pdf]

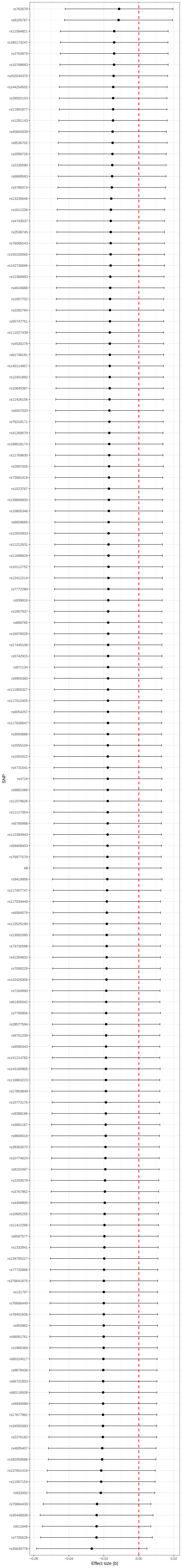
rs762679
rs8105767
rs11584821
rs185174247
rs2763979
rs10768683
rs202034370
rs144204502
rs28502153
rs11991877
rs1291143
rs45604339
rs6536702
rs2056726
rs2230590
rs6669563
rs3785074
rs13230646
rs1611236
rs4743037
rs2538745
rs76065543
rs150150565
rs142730696
rs12389950
rs4616688
rs1907702
rs2282764
rs55747751
rs111527438
rs4530278
rs61748181
rs145114957
rs12451892
rs10645387
rs11426156
rs6007020
rs76219171
rs41269079
rs188918174
rs11769630
rs2967355
rs73581419
rs1023767
rs139669835
rs10805346
rs6659669
rs12925933
rs11212631
rs11699829
rs10112752
rs12412214
rs7772289
rs939916
rs1957937
rs869785
rs16978028
rs17445108
rs5742915
rs871134
rs9955360
rs111950327
rs117512405
rs6054257
rs117630647
rs3093888
rs2555104
rs1003322
rs4731541
rs4724
rs6881568
rs11579626
rs11117354
rs6790988
rs112394943
rs59409453
rs79977579
All
rs9419958
rs117407747
rs117034449
rs6584579
rs113525195
rs13062095
rs73730598
rs41304832
rs7099229
rs142426306
rs7164950
rs61405042
rs7790856
rs28577594
rs6751209
rs6590343
rs141214782
rs143190905
rs116863223
rs17803849
rs10773176
rs9398196
rs3891167
rs9600019
rs28363070
rs10774624
rs8102497
rs2293579
rs3767952
rs4498805
rs10905255
rs11412296
rs6587577
rs1332941
rs139795227
rs77732866
rs376641875
rs131797
rs76666449
rs78491606
rs450962
rs56061761
rs1985369
rs80324517
rs9878436
rs66731853
rs80116508
rs9940099
rs17677991
rs34550383
rs2276182
rs4695407
rs182059586
rs137901416
rs11557154
rs932002
rs75664430
rs35446936
rs611646
rs7705526
rs35840778
SNP
-0.06
-0.04
-0.02
0.00
0.02
Effect size (b)
